# Human coronary plaque wall thickness correlated positively with flow shear stress and negatively with plaque wall stress: an IVUS-based fluid-structure interaction multi-patient study

**DOI:** 10.1186/1475-925X-13-32

**Published:** 2014-03-26

**Authors:** Rui Fan, Dalin Tang, Chun Yang, Jie Zheng, Richard Bach, Liang Wang, David Muccigrosso, Kristen Billiar, Jian Zhu, Genshan Ma, Akiko Maehara, Gary S Mintz

**Affiliations:** 1 School of Information and Communication Engineering, Beijing University of Posts and Telecommunications, Beijing, China; 2 School of Biological Science and Medical Engineering, Southeast University, Nanjing, China; 3 Mathematical Sciences Department, Worcester Polytechnic Institute, Worcester, MA, USA; 4 Network Technology Research Institute, China United Network Communications Co., Ltd., Beijing, China; 5 Mallinckrodt Institute of Radiology, Washington University, St. Louis, MO, USA; 6 Cardiovascular Division, Washington University School of Medicine, Saint Louis, MO, USA; 7 Department of Biomedical Engineering, Worcester Polytechnic Institute, Worcester, MA, USA; 8 Department of Cardiology, Zhongda Hospital, Southeast University, Nanjing 210009, China; 9 The Cardiovascular Research Foundation, Columbia University, New York, NY, USA

**Keywords:** Coronary, Fluid-structure interaction, Plaque rupture, Plaque progression, IVUS

## Abstract

**Background:**

Atherosclerotic plaque progression and rupture are believed to be associated with mechanical stress conditions. In this paper, patient-specific in vivo intravascular ultrasound (IVUS) coronary plaque image data were used to construct computational models with fluid-structure interaction (FSI) and cyclic bending to investigate correlations between plaque wall thickness and both flow shear stress and plaque wall stress conditions.

**Methods:**

IVUS data were acquired from 10 patients after voluntary informed consent. The X-ray angiogram was obtained prior to the pullback of the IVUS catheter to determine the location of the coronary artery stenosis, vessel curvature and cardiac motion. Cyclic bending was specified in the model representing the effect by heart contraction. 3D anisotropic FSI models were constructed and solved to obtain flow shear stress (FSS) and plaque wall stress (PWS) values. FSS and PWS values were obtained for statistical analysis. Correlations with p < 0.05 were deemed significant.

**Results:**

Nine out of the 10 patients showed positive correlation between wall thickness and flow shear stress. The mean Pearson correlation r-value was 0.278 ± 0.181. Similarly, 9 out of the 10 patients showed negative correlation between wall thickness and plaque wall stress. The mean Pearson correlation r-value was -0.530 ± 0.210.

**Conclusion:**

Our results showed that plaque vessel wall thickness correlated positively with FSS and negatively with PWS. The patient-specific IVUS-based modeling approach has the potential to be used to investigate and identify possible mechanisms governing plaque progression and rupture and assist in diagnosis and intervention procedures. This represents a new direction of research. Further investigations using more patient follow-up data are warranted.

## Introduction

**Figure 1:**
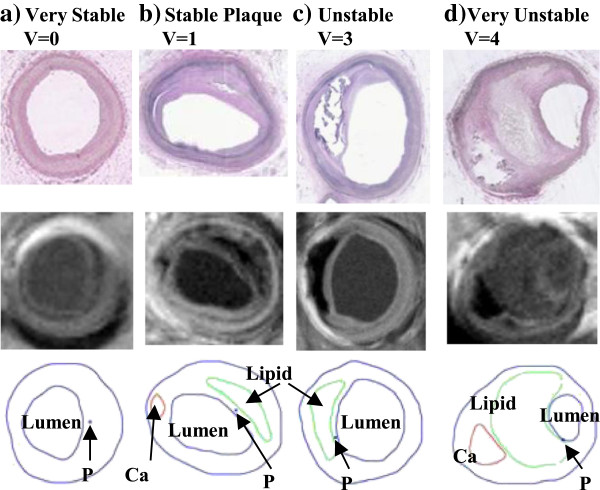
**Selected plaque samples with different vulnerability classified by histopathological analysis. (a)** Very stable plaque; **(b)** Stable plaque; **(c)** Unstable plaque; **(d)** Very unstable plaque. Each category included histology, MR image, and segmented contour plots, respectively.

Assessing atherosclerotic plaque vulnerability based on limited in vivo patient data has been a major challenge in cardiovascular research and clinical practice [1-7]. Considerable advances in medical imaging technology have been made in recent years to identify vulnerable atherosclerotic plaques in vivo with information about plaque components including lipid-rich necrotic pools, plaque cap, calcification, intraplaque hemorrhage, loose matrix, thrombosis, and ulcers, subject to resolution limitations of current technology. Atherosclerotic plaque progression and rupture are believed to be associated with mechanical stress conditions [6-18]. Parallel to histology-based atherosclerotic plaque classifications introduced by American Heart Association (AHA) [19-21], based on in vivo image data and computational modeling, we have introduced morphology- and stress-based plaque vulnerability indices which provide quantitative plaque assessment (Table 1 and Figure 1) [22]. Knowledge of those associations may be helpful for a better understanding of plaque progression and rupture process and for diagnosis and prevention of atherosclerosis-related cardiovascular diseases.

**Table 1:** Human coronary morphological plaque vulnerability index (MPVI) definition and AHA classifications

**MPVI**	**Plaque**	**Description**	**AHA classification**
V = 0	Very stable	Normal or slight intimal thickening	Type I, some atherogenic lipoprotein and intimal thickening
V = 1	Stable	Moderate intimal thickening, no extracellular lipid, calcification or significant inflammation	Type II (fatty streak), III (preatheroma)
V = 2	Slightly unstable	Small lipid core (<30% of plaque size); calcification may be present; thick fibrous cap (> 150 μm); little or no inflammation at plaque shoulders	Type IV, Vb, and Vc with less than 30% NC by area; or VII/VIII
V = 3	Moderately unstable	Moderate lipid core (30 – 40% of plaque size) and fibrous cap (65 – 150 μm); moderate intraplaque hemorrhage; moderate inflammation.	Type Va, IV/V with 30-40% NC by area
V = 4	Highly unstable	Large lipid core(>40%); thin fibrous cap (< 65 μm); large intraplaque hemorrhage; extensive inflammation; evidence of previous plaque rupture	Type VI; IV/V with > 40% NC by area

Cap thickness threshold values were modified from previously published values to adjust to the differences between coronary and carotid plaques based on available literature.

In vivo image-based coronary plaque modeling papers are relatively rare because clinical recognition of vulnerable coronary plaques has remained challenging [9,10,23]. We have published results based on follow-up studies showing that advanced carotid plaque had positive correlation with flow shear stress and negative correlation with plaque wall stress (PWS) [15]. In this paper, patient-specific intravascular ultrasound (IVUS)-based coronary plaque models with fluid-structure interaction (FSI), on-site pressure and ex vivo biaxial mechanical testing of human coronary plaque material properties were constructed to obtain flow shear stress and plaque wall stress data from ten (10) patients to investigate possible associations between vessel wall thickness and both flow shear stress and plaque wall stress conditions. The information may be helpful in establishing mechanisms governing plaque progression and rupture and may eventually be useful in cardiovascular disease diagnosis, prevention, or necessary interventions.

## Methods

### IVUS data acquisition

**Figure 2:**
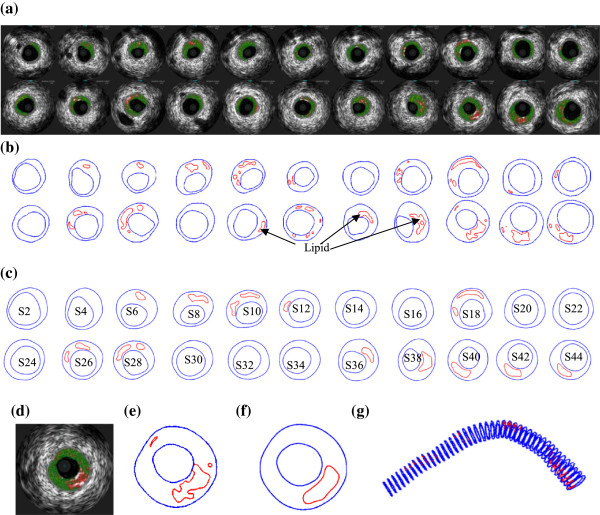
**IVUS model construction process.** Slice 1 is outlet. **(a)** Selected IVUS slices from 45-slice set; **(b)** Contour plots of selected IVUS slices from automated APIA segmentation; **(c)** Contour plots of selected IVUS slices after smoothing and used in FE model construction; **(d)** Enlarged view; **(e)** Enlarged contour; **(f)** Enlarged contour after smoothing; **(g)** 3D geometry showing 45 slices and lipid cores.

**Figure 3:**
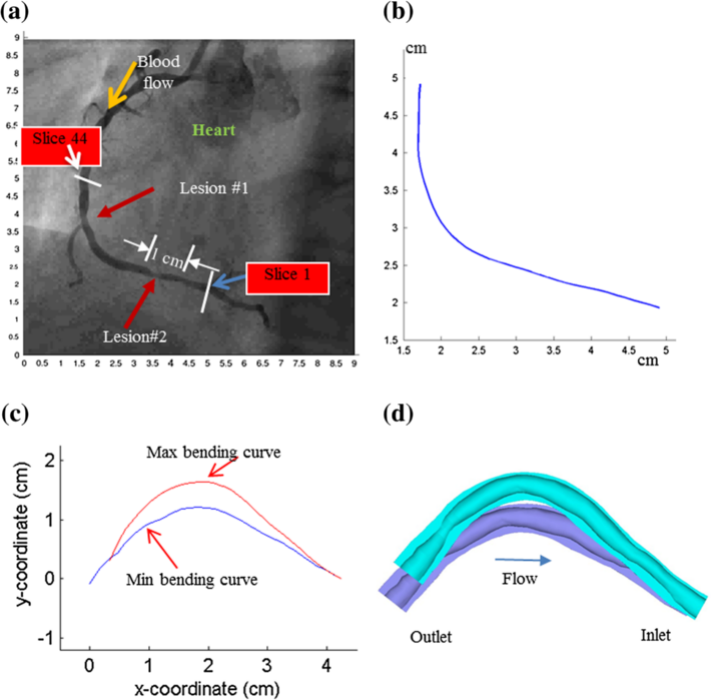
**X-Ray angiographic image, extracted centerlines of the coronary segment, and the re-constructed 3D geometry with maximum and minimum curvatures. (a)** X-Ray angiographic image; **(b)** Lumen center line extracted from X-Ray angiography; **(c)** Lumen center lines extracted from X-Ray angiography with max/min curvature; **(d)** Re-constructed 3D geometry with maximum and minimum curvatures. Flow direction is marked in **(a)**.

**Figure 4:**
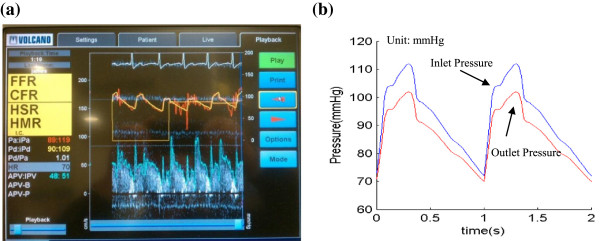
**Screen shoot from an IVUS Combo-Wire for patient-specific on-site blood pressure acquisition and pressure conditions using in the models. (a)** A snapshot of monitor screen showing on-site blood pressure and blood flow velocity; **(b)** Patient-specific blood pressure extracted from IVUS.

3D IVUS data were acquired during cardiac catheterization from 10 patients (7 M, 3 F, age: 48-75; median: 55) at Washington University at St. Louis (n = 6) and Cardiovascular Research Foundation (n = 4) after voluntary informed consent, using procedures described in Tang et al. [23]. In this paper, “plaque” was used to indicate the coronary vessel segment chosen for model construction and analysis. Plaque contour detection was performed using automated Virtual Histology software (ver. 3.1) on a Volcano s5 Imaging System (Volcano Corp., Rancho Cordova, CA). On-site blood pressure and flow velocity data were acquired using a Combo-Wire XT 9500 (Volcano Therapeutics, Inc.) 0.014-inch guide-wire with a Doppler flow velocity sensor. The X-ray angiogram (Allura Xper FD10 System, Philips, Bothel, WA) was obtained prior to the pullback of the IVUS catheter to determine the location of the coronary artery stenosis, vessel curvature and its cyclic bending caused by heart contraction. Figure 2 gives plots of a sample plaque IVUS slices, segmented contours, enlarged view, and the reconstructed 3D geometry showing lipid cores. The X-Ray angiogram and vessel bending were shown by Figure 3. Figure 4 shows an on-site pressure and flow velocity measurement screen shot and pressure condition digitized from the IVUS data.

### Biaxial testing and anisotropic model of human coronary material properties

**Figure 5:**
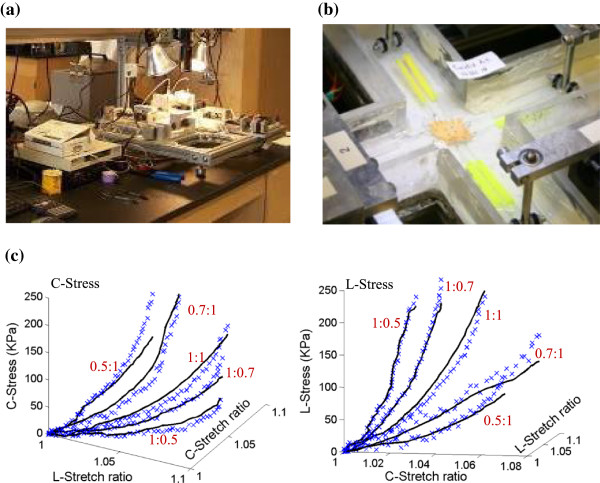
**The biaxial testing apparatus and sample results showing 3D plots of measured stress-stretch data from a human carotid sample with fitting curves by the anisotropic Mooney-Rivlin model. (a)** The biaxial testing apparatus; **(b)** Tissue sample mounted for biaxial test; **(c)** Anisotropic stress-stretch data from a human coronary sample.

A total of eight coronary arteries from 4 cadavers (age range: 50-81) were obtained from the National Disease Research Interchange, PA and from Washington University, St. Louis with proper consent. A custom planar biaxial test device was used under stress control to obtain stress and strain measurements over a wide range of ratios of stress along the longitudinal and circumferential axes of arterial specimen splayed open to form square samples (see Figure 5) [24]. The forces along the axes were measured via two torque transducers via rigid arms (effective resolution ~0.02 N) to determine the stress. Four graphite particles attached to the sample were tracked by a CCD camera to determine 2D strain (640 × 480 pixels; effective resolution ~0.07% strain). The applied maximum longitudinal: circumferential stress ratios were 1:1, 0.7:1, 0.5:1, 1:0.7 and 1:0.5. Based on average stress in the vessel walls at systolic pressure, the maximum engineering stress applied was 250 kPa. The vessel material was assumed to be hyperelastic, anisotropic, nearly-incompressible and homogeneous. A modified Mooney-Rivlin model was used to fit the biaxial data [23,24]:

(1) $$\begin{array}{ll} W= & c_{1}\left(I_{1}–3\right)+c_{2}\left(I_{2}–3\right)+D_{1}\left[\exp\left(D_{2}\left(I_{1}–3\right)\right)–1\right]\\ & +K_{1}/2K_{2}\left\{\exp\left[K_{2}{\left(I_{4}-1\right)}^{2}-1\right]\right\}. \end{array}$$

(2) $$I_{1}=\sum C_{\mathrm{ii}},I_{2}=½\left[{I_{1}}^{2}-C_{\mathrm{ij}}C_{\mathrm{ij}}\right],$$

where I_1_ and I_2_ are the first and second invariants of right Cauchy-Green deformation tensor **C** defined as ***C*** = [*C*_*ij*_] = **X**^T^**X**, **X** = [X_ij_] = [∂x_i_/∂a_j_], (x_i_) is current position, (a_i_) is original position, *I*_4_ = *C*_*ij*_(**n**_*c*_)_*i*_(**n**_*c*_)_*j*_*,***n**_*c*_ is the unit vector in the circumferential direction of the vessel, c_1_, D_1_, D_2_, and K_1_ and K_2_ are material constants. A least-squares method was used to determine the parameter values in Eq. (1) to fit our experimental circumferential and axial stress-stretch data [24]. Five human coronary plaque samples were tested and the one with median stiffness was used in this paper. The parameter values are: c_1_ = -1312.9 kPa, c_2_ = 114.7 kPa, D1 = 629.7 kPa, D_2_ = 2.0, K_1_ = 35.9 kPa, K_2_ = 23.5. Figure 5c shows that our model with parameters selected with this procedure fits very well with the measured experimental data. Our measurements are also consistent with data available in the literature [25-27].

### Reconstruction of plaque 3D geometry and mesh generation

All segmented 2D slices were read into ADINA input file. 3D plaque geometry was re-constructed following the procedure described in Yang et al. [23]. Because plaques have complex irregular geometries with component inclusions which are challenging for mesh generation, a component-fitting mesh generation technique was developed to generate mesh for our models [23]. Using this technique, the 3D plaque domain was divided into hundreds of small “volumes” to curve-fit the irregular plaque geometry with plaque component inclusions. The element type used for structural models (vessel and plaque components) was 3D solid 8-node element. The element type used for the fluid model was 3D fluid 4-node elements, free formed mesh. Mesh analysis was performed by decreasing mesh size by 10% (in each dimension) until solution differences (measured by L_2_ norms of solution differences of all components, including stress, strain, displacements, flow velocity, and pressure) were less than 2%. The mesh was then chosen for our simulations. The number of elements used for the 10 plaques is given in Table 2.

**Table 2:** Number of elements used in the 10 models

**Patient**	**Wall-element-tissue**	**Wall-element-lipid**	**Wall-element-Ca**	**Wall-all-element (sum)**	**Wall-node**	**Fluid-element**	**Fluid-node**
P1	18570	1554	0	20124	22880	62283	12027
P2	48840	1470	0	50310	55680	160752	29715
P3	22620	1140	0	23760	26800	108221	20226
P4	24504	888	0	25392	29914	227508	42312
P5	23976	1368	0	25344	29904	346195	61821
P6	19800	3240	0	23040	27216	291137	52262
P7	28071	279	0	28350	32000	195501	35764
P8	28122	228	0	28350	32000	192915	36017
P9	27148	2808	2384	32340	38073	209326	38273
P10	30164	320	1856	32340	38073	199434	36664

### The FSI model with cyclic bending and boundary conditions

3D anisotropic and isotropic multi-component FSI models were constructed to calculate flow and stress/strain distributions and evaluate the effects of cyclic bending and anisotropic properties and demonstrations were given using the plaque sample shown in Figure 2. Blood flow was assumed to be laminar, Newtonian, and incompressible. The Navier-Stokes equations with arbitrary Lagrangian-Eulerian formulation were used as the governing equations. Cyclic bending was specified by prescribing periodic displacement at the lower edge of the vessel using data obtained from X-Ray angiography and keeping the total length of the vessel unchanged. No-slip conditions and natural traction equilibrium conditions are assumed at all interfaces. With that, we have:

(3) $$\rho \left(\partial u/\partial t+\left(\left(u–u_{g}\right)\cdot \nabla \right)u\right)=-\nabla p+\mu \nabla^{2}u,$$

(4) ∇⋅u=0,

(5) $$u{\left|{}_{\Gamma }=\partial x/\partial t,\partial u/\partial n\right|}_{\mathrm{inlet},\mathrm{outlet}}=0,$$

(6) $$p{\left|{}_{\mathrm{inlet}}=p_{\mathrm{in}}\left(t\right),\;p\right|}_{\mathrm{outlet}}=p_{\mathrm{out}}\left(t\right),$$

(7) $$\rho \;v_{i,\mathrm{tt}}=\sigma_{\mathrm{ij},j},i,j=1,2,3;\mathrm{sum}\;\mathrm{over}\;j,$$

(8) $$\epsilon_{\mathrm{ij}}=\left(v_{i,j}+v_{j,i}+v_{\alpha ,i}v_{\alpha ,j}\right)/2,i,j,\alpha =1,2,3,$$

(9) $$\sigma_{\mathrm{ij}}\cdot n_{j}\left|{}_{\mathrm{out}\_\mathrm{wall}}\right)=0,$$

(10) $${\sigma^{r}}_{\mathrm{ij}}\cdot n_{j}{\left|{}_{\mathrm{interface}}={\sigma^{s}}_{\mathrm{ij}}\cdot n_{j}\right|}_{\mathrm{interface},}$$

where **u** and p are fluid velocity and pressure, **u**_g_ is mesh velocity, μ is the dynamic viscosity (μ = 0.04 P), ρ is density, Γ stands for vessel inner boundary, **x** is the current position of Γ, **σ** is stress tensor (superscripts indicate different materials), **ϵ** is strain tensor, **v** is solid displacement vector, superscript letters “r” and “s” were used to indicate different materials. For simplicity, all material densities were set to 1 g⋅cm^-3^ in this paper.

3D coronary plaque FSI models for the ten patients were constructed and solved by ADINA (Adina R &D, Watertown, MA) to calculate flow and stress/strain distributions. Each IVUS slice was divided into 4 quarters with each quarter containing 25 data points taken on the lumen. Average FSS and PWS values from each quarter were obtained from all slices of a plaque corresponding to maximum pressure condition for statistical analysis. Standard linear correlation analysis was performed to find possible correlations between wall thickness and the mechanical stressess (FSS and PWS). Correlations with p < 0.05 were deemed significant.

## Results

### Plaque wall thickness correlates positively with flow shear stress and negatively with plaque wall stress

**Figure 6:**
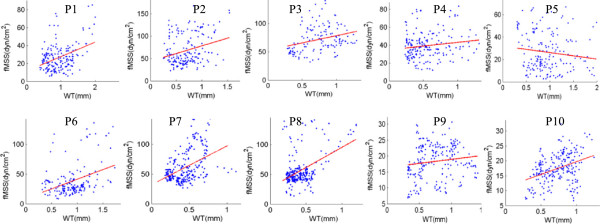
Mean-quarter flow shear stress vs. mean-quarter vessel wall thickness (WT) distribution plots from 10 patients showing positive correlation.

**Figure 7:**
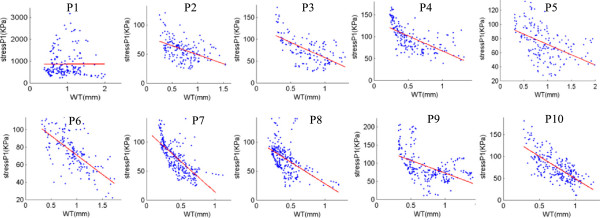
**Mean-quarter plaque wall stress (Stress-P**_**1**_**) vs. mean-quarter vessel wall thickness (WT) distribution plots from 10 patients showing negative correlation.**

Figures 6 &7 and Table 3 give the correlation results between plaque (wall) thickness and flow shear stress (FSS) and plaque wall stress (PWS), respectively. Mean quarter values were used in the analysis. Corresponding to maximum pressure condition (for simplicity, this is also when maximum curvature occurs), nine out of the 10 patients showed positive correlation between plaque wall thickness and flow shear stress. The mean Pearson correlation r-value was 0.278 ± 0.181. Similarly, 9 out of the 10 patients showed negative correlation between wall thickness and plaque wall stress. The mean Pearson correlation r-value was -0.530 ± 0.210.

**Table 3:** Plaque wall thickness correlates positively with flow shear stress and negatively with plaque wall stress

**Patient**	**P1**	**P2**	**P3**	**P4**	**P5**	**P6**	**P7**	**P8**	**P9**	**P10**
Segment length (cm)	2.15	5.16	3.30	6.60	4.40	2.80	3.15	3.15	27.5	27.5
MPVI	2	2	3	3	3	3	3	2	4	4
Qts	176	176	136	180	180	164	256	256	224	224
Correlation between vessel thickness and flow shear stress under maximum pressure										
r	0.3963	0.2842	0.2982	0.1501	-0.1305	0.377	0.443	0.456	0.1364	0.3687
p	0	0.0001	0.0004	0.0443	0.0808	0	0	0	0.0414	0
Correlation between vessel thickness and plaque wall stress under maximum pressure										
r	0.0019	-0.444	-0.614	-0.620	-0.477	-0.687	-0.714	-0.543	-0.502	-0.699
p	0.9799	0	0	0	0	0	0	0	0	0
Correlation between vessel thickness and flow shear stress under minimum pressure										
r	0.376	0.313	0.302	0.119	-0.151	0.378	0.459	0.436	0.124	0.368
p	0.000	0.000	0.000	0.113	0.042	0.000	0.000	0.000	0.063	0.000
Correlation between vessel thickness and plaque wall stress under minimum pressure										
r	0.007	-0.630	-0.712	-0.655	-0.487	-0.648	-0.811	-0.680	-0.379	-0.372
p	0.932	0.000	0.000	0.000	0.000	0.000	0.000	0.000	0.000	0.000

MPVI: Morphological plaque vulnerability index; Qts: Quarters.

Corresponding to minimum pressure condition (this is also when minimum curvature occurs), 7 out of the 10 patients showed positive correlation between plaque wall thickness and flow shear stress, 1 showed negative correlation, 2 showed no significance. The mean Pearson correlation r-value was 0.272 ± 0.189. For plaque wall stress, 9 out of the 10 patients showed negative correlation between wall thickness and plaque wall stress, about the same as the maximum pressure case. The mean Pearson correlation r-value was -0.537 ± 0.238.

### Effect of cyclic bending on plaque wall stress and strain behaviors

**Figure 8:**
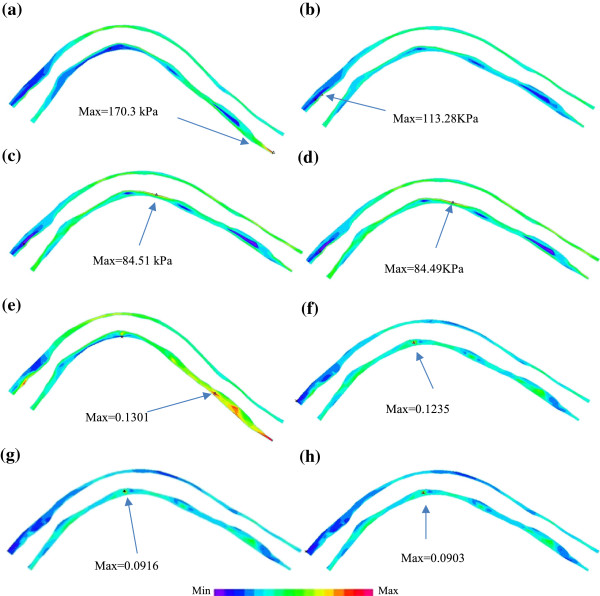
**Plots of plaque wall stress and strain from 2 models showing the effect of curvature variations on stress and strain distributions.** M1: baseline model with cyclic bending; M2: model using the minimum curvature without cyclic bending. **(a)** M1, PWS, max bending, Pin=112 mmHg; **(b)** M2, PWS, no cyclic bending, Pin=112 mmHg; **(c)** M1, PWS, min bending, Pin=72 mmHg; **(d)** M2, PWS, no cyclic bending, Pin=72 mmHg; **(e)** M1, PWSn, max bending, Pin=112 mmHg; **(f)** M2, PWSn, no cyclic bending, Pin=112 mmHg; **(g)** M1, PWSn, min bending, Pin=72 mmHg; **(h)** M2, PWSn, no cyclic bending, Pin=72 mmHg.

To demonstrate the effect of cyclic bending on plaque stress and strain behaviors, plaque wall stress (PWS) and strain (PWSn) from the baseline model of the plaque given in Figure 2 (Model 1-M1) and the model without cyclic bending (Model 2 – M2) are given in Figure 8. Model 2 used the minimum curvature from Model 1 and no cyclic bending was imposed. For simplicity, zero phase angle between pressure profile and curvature change was assumed, i.e., maximum and minimum bending in Model 1 occurred with maximum and minimum pressure conditions. Maximum PWS from M1 corresponding to maximum bending was about 100% higher than that corresponding to minimum bending (170.3 kPa vs. 84.51 kPa), while maximum PWS from M2 (no bending) corresponding to the maximum pressure condition was only 34% higher than that corresponding to minimum pressure condition (113.28 kPa vs. 84.49 kPa). Maximum PWS at maximum bending from M1 was about 50% higher than that from M1 at the same pressure condition. Differences in maximum of PWSn were similar to that of PWS. It should be noted that cyclic bending changed the stress/strain distribution patterns. The maximum bending caused some compression to the inner side of the vessel as shown by the lower PWS and PWSn regions in Figure 8a and e.

### Effects of cyclic bending on flow behaviors

**Figure 9:**
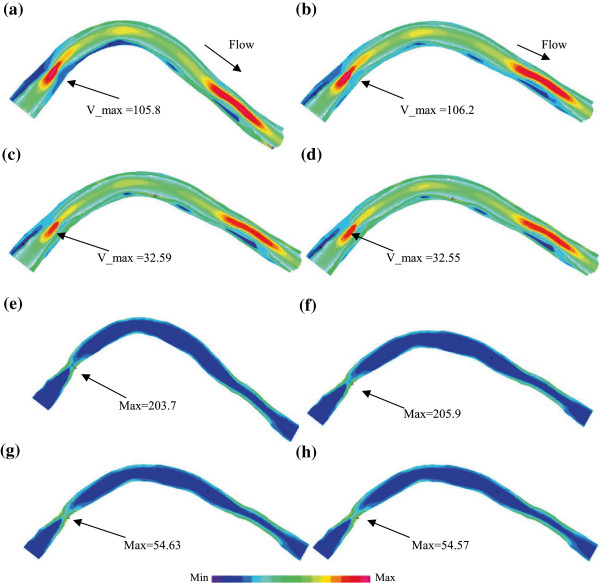
**Plots of flow velocity and flow shear stress from M1 and M2 showing the effect of curvature variations on stress and strain distributions.** M1 and M2 were defined as before. **(a)** M1, Velocity + PWS, max bending, Pin=112 mmHg; **(b)** M2, Velocity + PWS, no cyclic bending, Pin=112 mmHg; **(c)** M1, Velocity + PWS, min bending, Pin=72 mmHg; **(d)** M2, Velocity + PWS, No cyclic bending, Pin=72 mmHg; **(e)** M1, Flow shear stress, max bending, Pin=112 mmHg; **(f)** M2, Flow shear stress, no cyclic bending, Pin=112 mmHg; **(g)** M1, Flow shear stress, min bending, Pin=72 mmHg; **(h)** M2, Flow shear stress, no cyclic bending, Pin=72 mmHg.

It is reasonable to expect that cyclic bending would have impact on flow behaviors. Flow velocity and flow shear stress from M1 and M2 are given in Figure 9. It is observed that flow velocity and flow shear stress from both M1 and M2 corresponding to maximum pressure condition were much higher than those corresponding to minimum pressure condition. That was caused by flow rate changes in the cardiac cycle. Cyclic bending did cause about 1% (or less) decreases in flow velocity and flow shear stress. The decreases were smaller than previously reported [23] because the curvature change is small between M1 and M2 while we compared a curved vessel with a nearly straight vessel.

## Discussion

Our results show that IVUS data could be used to construct computational models to calculate flow shear stress and plaque stress/strain conditions which may be used to identify possible mechanisms governing plaque progression and rupture. This adds mechanical stress conditions into the list of risk factors and represents a new direction of research. While many factors are involved in plaque progression and rupture process, it is natural to think that final plaque rupture happens when critical plaque stress/strain exceed the plaque cap ultimate material strength. IVUS-based computational models can provide accurate stress/strain calculations and can serve as a useful tool for physicians in their diagnosis and intervention surgical decision making process.

It should be made clear that our current data is wall thickness, which is not progression by itself. We are currently working on patient follow-up data and will report our findings when available. Plaque progression and rupture are closely related to each other. A better understanding of plaque progression may lead to better understanding of plaque rupture process and more accurate plaque assessment schemes.

Some limitations of this study include: a) patient-specific and tissue-specific material properties were not available for our study; b) while the angiographic movie provided information for the position of the myocardium and partial information for curvature variations, two movies with different (preferably orthogonal) view angles are needed to re-construct the 3D motion of the coronary and provide accurate curvature variation information; c) some data such as zero-stress conditions (opening angle), multi-layer vessel morphology and material properties are not possible to measure non-invasively in vivo; d) tethering and interaction between the heart and vessel could not be included because those measurements are not currently available. A model coupling heart motion and coronary bending would be desirable when required data become available.

## Conclusion

Image-based computational models with cyclic bending and fluid-structure interactions could be used to provide more accurate flow and mechanical stress/strain calculations which may be useful for plaque assessment and identification of mechanisms governing plaque progression and rupture. Our results indicated that plaque wall thickness had positive correlation with flow shear stress and negative correlation with plaque wall stress. More patient follow-up data are needed to continue our investigations.

## Competing interests

Other than the grants listed in the acknowledgement section, the authors declare that they have no other competing interest.

## Authors’ contributions

DT, JZ and GSM were responsible for the design, data collection and overall investigation. RF, CY and WL were responsible for computational modeling and statistical data analysis. KB was responsible for plaque material mechanical testing. JZ, DM, JZ, GM, AM and GSM were responsible for data collection and image analysis. All authors 1) have made substantial contributions to conception and design, or acquisition of data, or analysis and interpretation of data; 2) have been involved in drafting the manuscript or revising it critically for important intellectual content; and 3) have given final approval of the version to be published. Each author has participated sufficiently in the work to take public responsibility for appropriate portions of the content.

## Authors’ information

Tang’s group has been publishing image-based modeling work in recent years. For more information, please visit Tang’s website: http://users.wpi.edu/~dtang/.

The Washington University group (Jie Zheng and Richard Bach) has been publishing in medical imaging for vulnerable plaques extensively, see website:

http://www.mir.wustl.edu/research/physician2.asp?PhysNum=78, and

http://wuphysicians.wustl.edu/physician2.aspx?PhysNum=2687

Ma and Zhu are clinicians and have been doing research in interventional medicine for coronary diseases: http://www.njzdyy.com/s/21/t/2/00/d2/info210.htm;

The Columbia group (Cardiovascular Research Foundation, Mintz and Maehara) has been playing a leading role in the cardiovascular research. Web: http://www.crf.org/
